# The effects of blood flow restriction training on early muscle strength and mid-term knee function following anterior cruciate ligament reconstruction: a systematic review and meta-analysis

**DOI:** 10.1186/s13018-025-05673-8

**Published:** 2025-03-13

**Authors:** Xiaoyan Li, Fajun Xiao, Hongying Ren, Yi Peng, Fang Feng, Qinjian Dong

**Affiliations:** 1 Department of Rehabilitation Medicine, Beijing Anzhen Nanchong Hospital, Capital Medical University & Nanchong Central Hospital, Nanchong, Sichuan Province China; 2 Department of Rehabilitation Medicine, Nanchong Mental Health Center of Sichuan Province, Nanchong, Sichuan Province China; 3 Chengdu University of Traditional Chinese Medicine, Chengdu, Sichuan Province 610075 China

**Keywords:** Blood flow restriction training, Anterior cruciate ligament reconstruction, Muscle strength, Knee function, Meta-analysis

## Abstract

**Objective:**

Early restoration of muscle strength and knee joint function after anterior cruciate ligament reconstruction (ACLR) is a critical goal in the rehabilitation process. Blood flow restriction training (BFRT), a low-load training method, has gained attention in musculoskeletal rehabilitation in recent years, but its specific effects in ACLR rehabilitation remain unclear.

**Methods:**

Relevant literature up to December 20, 2024, was searched in the PubMed, Embase, Cochrane, and Web of Science databases, and study selection was performed according to PRISMA guidelines. Randomized controlled trials (RCTs) and quasi-RCTs comparing the effects of BFRT and traditional training in ACLR rehabilitation were included. Data on early muscle strength (ACSA and MVIC) and mid-term knee function (IKDC scores and isometric strength of knee extensors) were extracted. The quality of the studies was assessed using the Cochrane risk of bias tool, and statistical analyses were conducted using fixed-effect or random-effect models.

**Results:**

A total of 11 studies involving 276 patients were included, with 139 in the BFRT group and 137 in the control group. Meta-analysis showed no significant improvements in quadriceps ACSA (SMD = 0.82, 95% CI: -0.17 to 1.81, *p* = 0.10) or MVIC (SMD = 0.47, 95% CI: -0.16 to 1.10, *p* = 0.15) during the early postoperative period (≤ 3 weeks). At mid-term follow-up (8–14 weeks), BFRT significantly improved IKDC scores (SMD = 3.70, 95% CI: 0.20 to 7.21, *p* = 0.04). No significant differences were observed between the groups in the improvement of isometric strength of knee extensors (SMD = 0.50, 95% CI: -0.62 to 1.63, *p* = 0.38).

**Conclusions:**

BFRT demonstrated limited effectiveness in early muscle strength recovery during ACLR rehabilitation but may have a positive impact on mid-term knee function, particularly in improving IKDC scores. However, due to heterogeneity and potential bias in the included studies, future research should incorporate more high-quality, multicenter RCTs to further validate the mid- to long-term value of BFRT in postoperative rehabilitation.

## Introduction

The anterior cruciate ligament (ACL) is a crucial structure in the knee joint, primarily responsible for restricting excessive anterior translation and internal rotation of the tibia relative to the femur, thereby maintaining knee joint stability [1, 2]. ACL injury is one of the most common knee injuries in sports medicine, often leading to knee instability and functional impairments that can severely affect athletic performance and quality of life [3–5]. Annually, over 2 million ACL injuries of varying severity are reported worldwide [6]. For complete ACL ruptures, surgical reconstruction is typically required to restore knee function [7–9]. Currently, ACL reconstruction (ACLR) is the standard treatment for ACL tears, aiming to restore knee stability and function [10, 11]. However, patients frequently encounter significant challenges in regaining muscle strength and improving knee function post-surgery, making the rehabilitation process both complex and essential [12, 13].

In ACLR rehabilitation, traditional high-load resistance training may increase postoperative joint stress, potentially hindering tissue healing [14]. Additionally, due to postoperative pain and tissue edema, patients often struggle to engage in timely, effective, and sufficiently intense rehabilitation, which frequently delays the recovery of knee function [15]. Blood flow restriction training (BFRT) is a method that promotes muscle strength and hypertrophy under low-load conditions by applying appropriate blood flow restriction [16]. In recent years, the clinical application of BFRT has become increasingly widespread, demonstrating positive effects in musculoskeletal rehabilitation. Studies have shown that combining BFRT with low-intensity resistance training can significantly improve muscle strength and endurance while reducing joint stress associated with high-load training and lowering the risk of injury [17, 18]. Furthermore, BFRT is thought to enhance muscle protein synthesis, thereby enhancing improving function. The mechanisms of BFRT may involve increased metabolic stress, altered muscle fiber recruitment patterns, and the promotion of endogenous growth factors such as insulin-like growth factor 1 (IGF-1) [19, 20]. As a low-load, highly efficient training method, BFRT offers a promising and safe rehabilitation strategy for patients following ACLR.

Previous meta-analyses have mainly focused on the effects of BFRT on postoperative muscle strength and volume in ACLR patients [21], without conducting phase-specific analyses based on follow-up periods. Additionally, a meta-analysis in 2023 found no significant difference between BFRT and regular training in improving or maintaining thigh muscle size and flexion-extension strength [22]. Due to the heterogeneity in BFRT training protocols and the timing of outcome measurements, the overall effectiveness of BFRT for ACLR patients remains uncertain. Systematic studies and definitive conclusions regarding the stage-specific effects of BFRT on early muscle strength and mid-term knee function in ACLR rehabilitation remain insufficient. Existing research indicates that ACLR patients often experience disuse atrophy due to the inability to tolerate traditional rehabilitation training during the early postoperative period. Within the first three weeks after surgery, patients may lose approximately 20–33% of quadriceps muscle volume, and these adverse effects can persist for years [23, 24].

The present meta-analysis aims to assess the effects of BFRT on early muscle strength and mid-term knee function after ACLR through a systematic review and meta-analysis, stratified by postoperative follow-up duration. This study seeks to provide evidence-based guidance for ACLR rehabilitation and offer a scientific reference for optimizing clinical rehabilitation strategies.

## Materials and methods

### Search strategy

We conducted a literature search through PubMed, Embase, Cochrane, and Web of Science databases, covering the period from inception to December 20, 2024. The search strategy was defined based on Medical Subject Headings (MeSH) terms: (“anterior cruciate ligament reconstruction” OR “anterior cruciate ligament”) AND (“blood flow restriction therapy” OR “blood flow restriction” OR “blood flow restriction training” OR “BFRT”). In addition to the database, we also reviewed the full texts and references of studies that met the inclusion criteria to ensure the inclusion of relevant research. This meta-analysis was conducted in accordance with the Preferred Reporting Items for Systematic Reviews and Meta-Analyses (PRISMA) guidelines [25]. The study protocol was registered in the International Prospective Register of Systematic Reviews under registry code CRD42025638939.

### Study selection

Two authors independently carried out the study selection process, which consisted of three main steps: removal of duplicate studies, preliminary screening based on titles and abstracts, and a more detailed screening based on full-text articles. Any disagreements regarding the final inclusion of studies were resolved through discussion with the involvement of a third author.

### Inclusion criteria and exclusion criteria

#### Inclusion criteria

(1) Participants aged 14–60 years with primary ACLR, and no restrictions on preoperative activity level or graft type (semitendinosus-gracilis, bone-patellar tendon-bone, or hamstring tendon); (2) The experimental group received BFRT therapy, while the control group received conventional rehabilitation without the use of BFRT; (3) The included study types were randomized controlled trials (RCTs), cohort studies, and case-control studies; (4) Studies published in English; (5) The study must report at least one clinical outcome, such as anatomical cross-sectional area (ACSA), maximal volitional isometric contraction (MVIC) torque, IKDC score, or knee extensor isometric strength.

#### Exclusion criteria

(1) Reviews, meta-analyses, abstracts, case reports, and non-controlled clinical studies; (2) Preclinical studies, including those conducted at the cellular, animal, or cadaver levels; (3) Studies involving patients with other musculoskeletal conditions that could affect the analysis results, such as severe ligament tears, meniscus injuries, or fractures; (4) Patients with concomitant meniscal repair requiring restricted weight-bearing; (5) Studies that did not report outcomes of interest.

### Data extraction and outcomes of interest

Two independent authors carefully reviewed and analyzed the final included studies and extracted basic information. The extracted details included: year of publication, first author, country, study type, patient age, gender, height, weight, BMI, number of cases, follow-up duration, and specific details of BFRT (occlusion tool, occlusion area, and intervention duration). The outcomes of interest primarily included early muscle strength evaluations (ACSA and MVIC torque) and mid-term knee joint function assessments (IKDC score, and knee extensor isometric strength). Any discrepancies in the data extracted by the two authors were resolved through discussion with a third author. Due to the inconsistency in the measurement time points of the outcome indicators, we performed a phase-based analysis of the included studies according to the stages outlined in the ACLR postoperative rehabilitation guidelines. The analysis includes the following two phases: (1) Early muscle strength (≤ 3 weeks): While protecting the graft and reducing swelling, emphasis is placed on rebuilding muscle strength; (2) Mid-term knee function (8–14 weeks): Continuing to protect the graft while maintaining full joint range of motion.

### Methodological quality assessment

The included studies were categorized as RCTs and quasi-RCTs, and their quality was assessed using the Cochrane Risk of Bias tool [26]. The evaluation covered five domains: selection bias (random sequence generation and allocation concealment), performance bias (blinding of participants and personnel, blinding of outcome assessment), attrition bias (incomplete outcome data), and reporting bias (selective reporting). The risk of bias was classified as “low risk”, “high risk”, or “unclear risk”. The assessment process was conducted independently by two authors, and any disagreements were resolved through discussion with a third author.

### Statistical analysis

Meta-analysis was conducted by extracting the mean values and standard deviations of postoperative outcome measures to estimate the standardized mean difference (SMD) and 95% confidence intervals (CI). Heterogeneity was assessed and quantified using the *χ*² test and *I*² statistics. Specifically, when *I*² ≤ 50% and *p* > 0.10, heterogeneity was considered low, and a fixed-effects model was applied for analysis; otherwise, a random-effects model was used [27]. Data presented in graphical form were extracted using Origin 2024 for further analysis. Statistical analysis was performed using RevMan 5.3 software (Cochrane Collaboration, Copenhagen, Denmark), and *p* < 0.05 was considered statistically significant.

## Results

### Literature search and screening

**Fig. 1:**
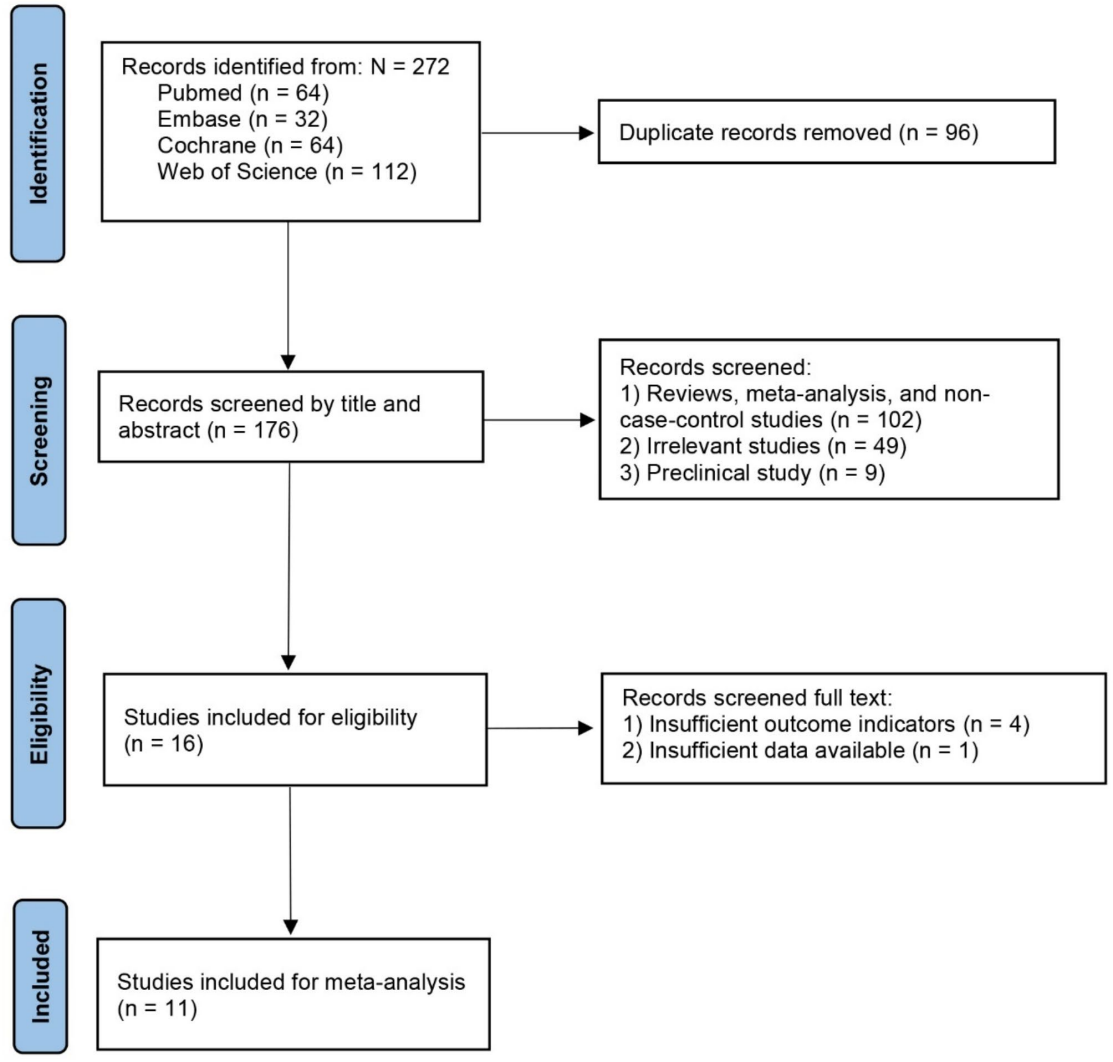
Flowchart of the study based on the PRISMA guidelines

A comprehensive search of the PubMed, Embase, Cochrane, and Web of Science databases yielded a total of 272 potential studies. Specifically, 96 duplicate studies were removed first, followed by the exclusion of 160 studies based on their titles and abstracts. Finally, 5 studies were excluded after full-text review due to the lack of outcome measures or inability to extract data, leaving 11 studies [28–38] included for subsequent analysis. The study selection process is illustrated in Fig. 1.

### Study characteristics

A total of 276 patients were included across the 11 studies, with 139 patients receiving BFRT treatment and 137 patients undergoing conventional rehabilitation. The mean age ranged from 14.8 to 50.9 years, and the average body mass index (BMI) ranged from 19.6 to 32.2. In four studies, patients underwent ACLR using autologous semitendinosus-gracilis grafts, while two studies used bone-patellar tendon-bone and hamstring tendon grafts, respectively. One study included patients who underwent three different reconstruction techniques, while the remaining four studies did not report the graft type. Three studies reported concurrent surgeries performed alongside ACLR. All studies described the blood flow restriction tools and occlusion areas used, with the occlusion region being the proximal thigh in all cases. In terms of study design, seven studies were RCTs, and four were quasi-RCTs. Regarding outcome measures, early outcomes (≤ 3 weeks) included ACSA and MVIC torque, while mid-term outcomes (8–14 weeks) included IKDC score, and knee extensor isometric strength. The baseline characteristics of the included patients and details of BFRT are shown in Table 1.

**Table 1:** Characteristics of articles included in the meta-analysis

Author	Year	Country	Study design	Age (BFRT vs. Control, years ± SD)	Number (BFRT vs. Control)	Baseline Characteristics (Height/Weight/BMI, BFRT group)	Baseline Characteristics (Height/Weight/BMI, Control group)	Graft Origin	Occlusion Tool (Cuff Width)	Concomitant Surgical Procedures	Occlusion Area	Pressure Level	Intervention Duration	Comparison Group	Exercise	Frequency	Total time	Duration
Iversen et al.	2016	Norway	RCT	24.9 ± 7.4 vs. 29.8 ± 9.3	12 vs. 12	176.9 ± 7.9/76.9 ± 12.1/NA	178.9 ± 7.8/77.6 ± 9.6/NA	hamstring tendon graft (HS)	Delfi low pressure cuff (14 cm width)	/	most proximal part of the thigh	130-180mmHg	day 2–14 after surgery	non-BFR	Isometric quadriceps contractions, leg extension over a knee-roll, straight leg-raises	2/day	12 days	restriction of blood flow 5 min, 5 groups and 20 repetitions per group
Zargi et al.	2016	Slovenia	Quasi-RCT	33 ± 7 vs. 34 ± 10	10 vs. 10	NA/NA/23.7 ± 3.1	NA/NA/23.5 ± 3.9	semitendinosus-gracilis autograft	VariFit Conture Thigh Cuff (14 cm width)	12 meniscectomy	most proximal part of the thigh	150 mmHg	10 days before surgery	sham-BFR at 20 mmHg	unilateral resisted knee extension	3/week	9 days	restriction of blood flow, 6 groups and 40 repetitions per group
Zargi et al.	2018	Slovenia	Quasi-RCT	34 ± 6 vs. 35 ± 5	10 vs. 10	NA/NA/24.3 ± 3.9	NA/NA/23.9 ± 2.9	semitendinosus-gracilis autograft	VariFit Conture Thigh Cuffs(14 cm width)	12 meniscectomy	most proximal part of the thigh	150 mmHg	8 days before surgery	sham-BFR at 20 mmHg	unilateral resisted knee extension	3/week	1 week	restriction of blood flow, 6 groups and 40 repetitions per group
Takarada et al.	2000	Japan	RCT	22.4 ± 2.1 vs. 23 ± 2.5	8 vs. 8	NA/NA/NA	NA/NA/NA	/	Pneumatic occlusion cuff(90 mm width)	/	most proximal part of the thigh	200–260 mmHg	3 days before surgery	placebo occlusion	/	2/day	2 weeks	restriction of blood flow for 5 min, 5 groups of exercise
Kacin et al.	2021	Slovenia	Quasi-RCT	38 ± 6 vs. 38 ± 8	6 vs. 6	NA/NA/26.0 ± 4.9	NA/NA/ 25.8 ± 5.4	semitendinosus-gracilis autograft	Ischemic Trainer double-chamber cuff (13.5 cm width)	/	most proximal part of the thigh	150 mmHg	before surgery	sham-BFR at 20 mmHg	Knee flexion and extension of the injured leg.	3/week	3 weeks	restriction of blood flow, 4 groups and 40 repetitions per group
Jung et al.	2022	Korea	Quasi-RCT	30.83 ± 7.59 vs. 27.83 ± 8.43	12 vs. 12	170.27 ± 8.80/78.68 ± 18.63/NA	172.58 ± 4.76/71.98 ± 10.05/NA	/	Smart Tool Plus single-chamber pneumatic cuff	/	most proximal part of the thigh	40% systolic blood pressure	3 days after surgery	non-BFR	enhance range of motion (ROM exercise), weight-bearing exercise, closed kinetic chain (CKC) exercise and open kinetic chain (OKC) exercise	3/week	12 weeks	restriction of blood flow, Four sets of exercises and with each set resting for 30 s, totaling 75 times
Li et al.	2023	China	RCT	30.50 ± 5.26 vs. 28.33 ± 5.19	8 vs. 6	175.87 ± 11.06/79.13 ± 12.78/NA	172.33 ± 12.56/74.67 ± 23.09/NA	/	AirBands	/	proximal thigh	80% Arterial occlusion pressure	> 8 weeks after surgery	non-BFR	elastic band, yoga ball, self-weight lunge squat, up and down stairsnge squat, resistance squat, single-leg Bulgarian squat, resistance lunge walk, Thera-Band USA elastic bands, utilized red ones.	2/week	8 weeks	restriction of blood flow, Four sets of exercises and with each set resting for 30 s, totaling 75 times
Jack II et al.	2022	USA	RCT	28.1 ± 7.4 vs. 24.1 ± 7.2	17 vs. 15	173.6 ± 9.2/76.6 ± 15.5/25.2 ± 2.8	170.9 ± 12.4/79.3 ± 22.0/26.9 ± 5.3	bone-patellar tendon-bone (BPTB)	Delfi Medical	/	most proximal part of the thigh	80% arterial limb occlusion pressure	7 days after surgery	non-BFR	Quadriceps Contractions, Closed-chain Knee Extensions, Bilateral Leg Press, Single Leg Press, Single Leg Hamstring Curl, Ball Squats, Split Lunges, Box Step-ups	2/week	12 weeks	restriction of blood flow, Four sets of exercises and with each set resting for 30 s, totaling 75 times
De Melo et al.	2022	Brazil	RCT	41.1 ± 9.8 vs. 39.6 ± 10.8	12 vs. 12	173.1 ± 7.6/73.1 ± 13.9/24.2 ± 3.0	170.2 ± 6.2/68.6 ± 10.4/23.6 ± 2.4	/	Cuff Scientific Leg^®^–WCS	/	most proximal part of the thigh	80% arterial limb occlusion pressure	3 days after surgery	non-BFR	Leg press, Flexor chair	2/week	12 weeks	restriction of blood flow, Four sets of exercises and with each set resting for 30 s, totaling 75 times
Ohta et al.	2003	Japan	RCT	28 ± 9.7 vs. 30 ± 9.7	22 vs. 22	NA/65 ± 14/NA	NA/63 ± 8.8/NA	semitendinosus–gracilis tendon	Hand-pumped tourniquet	/	proximal part of the thigh	180 mmHg	2 weeks after surgery	non-BFR	Straight leg raising, hip joint abduction, Half-squat, Step-up, Elastic tube, Knee-bending walking	6/week	14 weeks	restriction of blood flow, 1–3 groups and 20 repetitions per group
Okoroha et al.	2023	USA	RCT	25.4 ± 10.6 vs. 27.5 ± 12	22 vs. 24	NA/NA/24.9 ± 3.1	NA/NA/26.9 ± 5.3	30 BPTB,13 HS,3 quadriceps tendon autograft	Single-chamber pneumatic torniquet (Smart Tool Plus)	9 meniscal repair,18 meniscectomy	most proximal part of the thigh	80% limb occlusion pressure	2 weeks before surgery	non-BFR	quadriceps contractions in end-range extension, straight-leg raises, long-arc quadriceps sets, and quarter squats.	5/week	14 weeks	restriction of blood flow, Four sets of exercises and with each set resting for 30 s, totaling 75 times

### Quality assessment

**Fig. 2:**
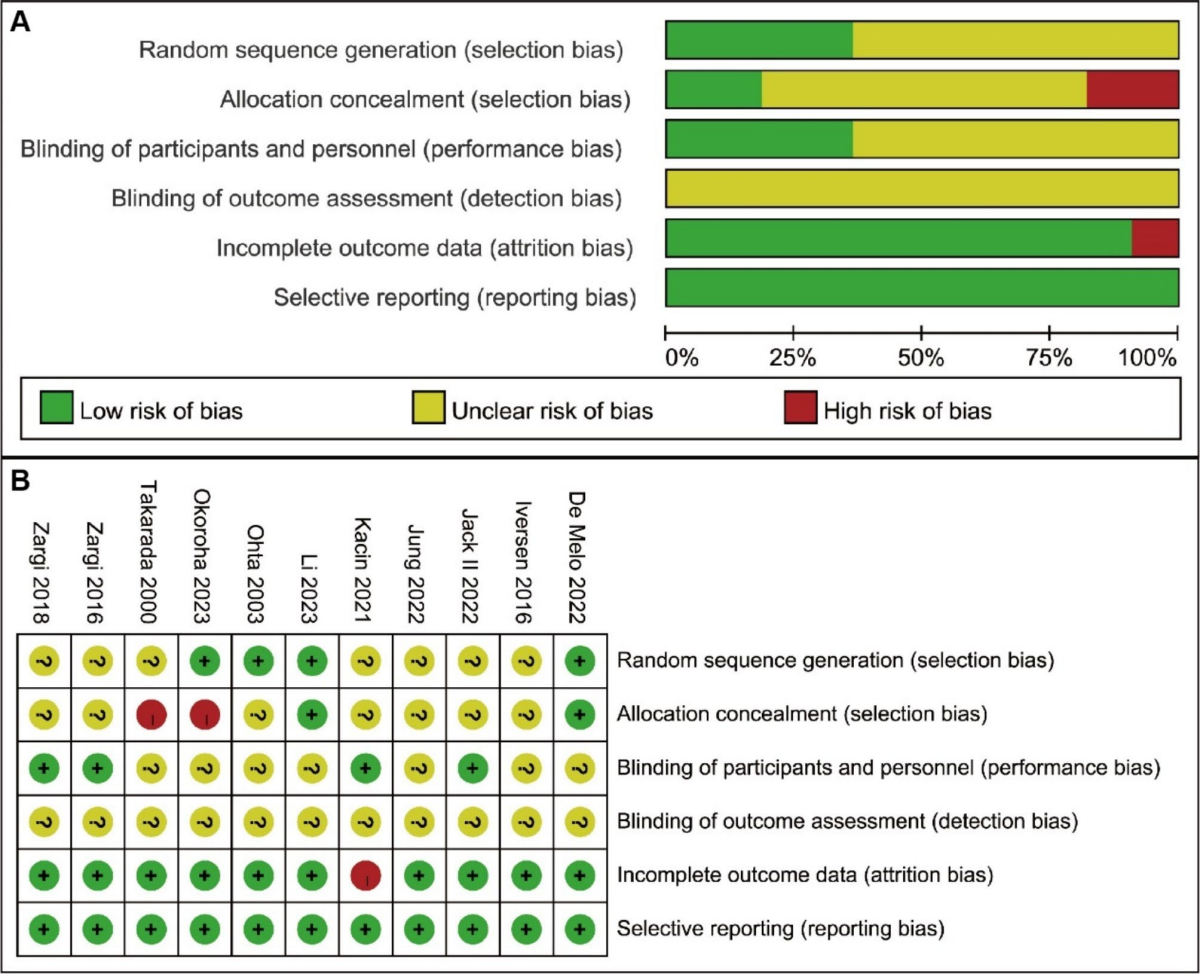
Summary results of the risk of bias assessment. (**A**) Overall risk of bias for each type, presented as proportions of low risk (green), unclear risk (yellow), and high risk (red). (**B**) Risk of bias assessments for each individual study across different types of bias

Following independent evaluation and summary by two authors, the risk of bias assessment for the 11 included studies is shown in Fig. 2A and B. For selection bias, four studies provided detailed descriptions of the methods used to generate allocation sequences, and two studies reported methods for concealing allocation sequences. Two studies explicitly stated that patients were aware of their group assignments, while the remaining studies were classified as having unclear risk of bias. For performance bias, four studies reported the use of blinding for both implementers and patients, while the rest were considered to have an unclear risk of bias. All studies were assessed as having an unclear risk of bias for detection bias in outcome measurements. Additionally, only one study was judged to have a high risk of attrition bias due to loss to follow-up, while the remaining studies were classified as having low risk of attrition bias. Overall, most studies were assessed as having low or unclear risk of bias, and the general quality of the included studies was deemed acceptable.

### Clinical efficacy

#### Early muscle strength (≤ 3 weeks)

**Fig. 3:**
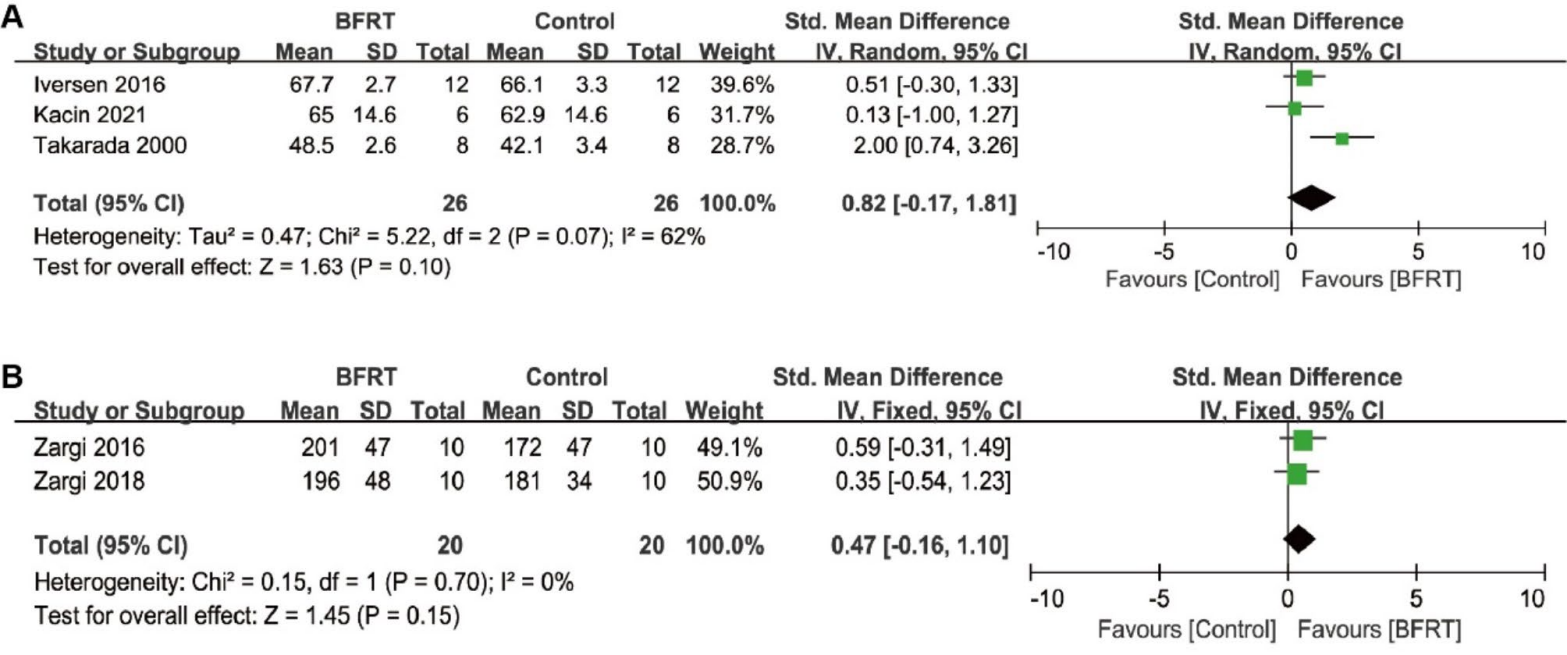
Forest plot comparing early muscle strength (≤ 3 weeks) between the BFRT group and the control group. (**A**) anatomical cross-sectional area (ACSA); (**B**) maximal volitional isometric contraction (MVIC) torque

The recovery of early muscle strength is crucial for the prognosis of patients after ACLR surgery. A total of five studies reported the effects of BFRT on early muscle strength, with three studies investigating the quadriceps anatomical cross-sectional area (ACSA, cm²) and two studies evaluating muscle maximal volitional isometric contraction (MVIC) torque (Nm). For quadriceps ACSA, due to significant heterogeneity (*p* = 0.07, *I*² = 62%), a random-effects model was applied for pooled analysis. The results indicated that BFRT did not demonstrate early improvement in quadriceps volume compared to conventional non-BFRT training (SMD = 0.82; [95% CI, -0.17 to 1.81]; *p* = 0.10) (Fig. 3A). Similarly, the pooled analysis of two studies showed no beneficial effect of BFRT on early muscle strength improvement (SMD = 0.47; [95% CI, -0.16 to 1.10]; *p* = 0.15) (Fig. 3B).

#### Mid-Term knee function (8–14 weeks)

**Fig. 4:**
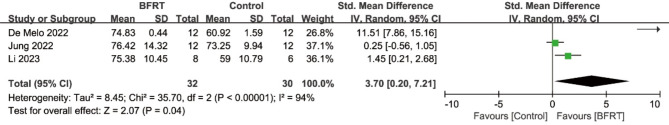
Forest plot comparing IKDC score (8–14 weeks) between the BFRT group and the control group

Additionally, five studies assessed mid-term knee function outcomes in both groups during follow-up. Among them, three studies investigated the IKDC score. Specifically, the pooled analysis of three studies (a total of 62 patients) on IKDC scores showed significant heterogeneity (*p* < 0.00001, *I*² = 94%). Using a random-effects model, the analysis indicated that BFRT significantly improved mid-term knee function compared to conventional non-BFRT training (SMD = 3.70; [95% CI, 0.20 to 7.21]; *p* = 0.04) (Fig. 4). However, due to the high heterogeneity of the pooled analysis, this result should be interpreted cautiously.

#### Mid-Term muscle strength (8–14 weeks)

**Fig. 5:**
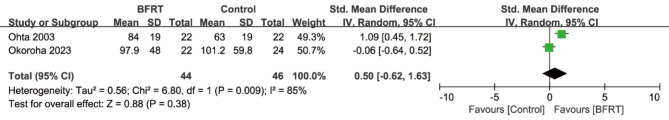
Forest plot comparing knee extensor isometric strength (8–14 weeks) between the BFRT group and the control group

Two studies reported knee extensor isometric strength, reflecting the mid-term recovery of muscle strength in patients. Due to significant heterogeneity (*p* = 0.009, *I*² = 85%), a random-effects model was applied for the pooled analysis. The results indicated that BFRT did not show an improvement in mid-term muscle strength recovery compared to conventional non-BFRT training (SMD = 0.50; [95% CI, -0.62 to 1.63]; *p* = 0.38) (Fig. 5).

## Discussion

ACLR is the primary surgical approach for treating ACL tears, with the goal of restoring knee stability and function [39, 40]. However, muscle weakness and joint dysfunction during the postoperative rehabilitation process continue to pose significant challenges [40–42]. Recently, BFRT, which combines low-load resistance training with blood flow restriction, has gained attention for its potential to enhance muscle strength and promote functional recovery [17]. The application of BFRT in ACLR rehabilitation offers a promising solution to accelerate muscle strength recovery and improve knee function. This study systematically evaluated the effects of BFRT on early muscle strength and mid-term knee function following ACLR.

This study found that during the early postoperative period (≤ 3 weeks) following ACLR, BFRT did not demonstrate significant advantages in improving the quadriceps anatomical cross-sectional area (ACSA) or maximum voluntary isometric contraction (MVIC). Early postoperative inflammatory responses may limit muscle strength recovery. However, contrary to our findings, some studies suggest a potential role for BFRT in early muscle strength recovery. For example, Brandner et al. [43] reported that BFRT significantly improved knee extension strength at 4 weeks. During the earlier period of 1–2 weeks, the BFRT group experienced more rapid muscle mass gains [44]. Karabulut et al. [45] found that BFRT under low-load conditions could significantly enhance serum bone turnover markers, promoting bone formation. These discrepancies may arise from variations in intervention parameters across studies, such as BFRT pressure intensity, training frequency, and the initial muscle condition of postoperative patients. Heterogeneity analysis revealed considerable heterogeneity (*I*² = 62%) in early muscle strength recovery, potentially influenced by differences in patient characteristics and the timing of interventions. Future research should focus on standardizing intervention parameters, such as specifying the pressure range (e.g., 40-80% of arterial occlusion pressure) and training frequency, to reduce variability in study outcomes.

At mid-term follow-up (8–14 weeks), this study found that BFRT exhibited a positive effect on improving knee function (IKDC score) (SMD = 3.70, *p* = 0.04), while its impact on isometric strength of the knee extensors was not significant. This suggests that BFRT may be more effective for enhancing subjective functional evaluations and dynamic knee stability. The improvement in IKDC scores may be attributed to the enhancement of local muscle oxidative metabolic capacity facilitated by BFRT. By partially restricting blood flow, BFRT increases metabolic stress levels and promotes the release of endogenous growth factors, such as IGF-1, which facilitate soft tissue repair and adaptive muscle strengthening [46]. Additionally, previous studies have shown that BFRT can promote skeletal muscle hypertrophy and strength enhancement through various mechanisms, including increased hormone levels, activation of the mTOR pathway to stimulate protein synthesis, and the promotion of satellite cell activity (Mechanisms Behind Blood Flow-Restricted Training and its Effect Toward Muscle Growth). This perspective is supported by Hughes et al. [47], who found that BFRT combined with low-load training helps improve physical function and quality of life.

The recovery of isometric knee extensor strength may be influenced by the postoperative tissue healing process, and the duration of BFRT intervention may not have been sufficient to demonstrate its effects. Furthermore, the high heterogeneity observed (e.g., *I*² = 94% for IKDC scores) suggests that variations in rehabilitation protocols and patient characteristics across studies may have significantly influenced the outcomes. In some studies, patients underwent concurrent surgeries or utilized different reconstruction techniques, which could affect the rate of knee function recovery. These factors highlight the need for standardized protocols and careful consideration of patient-specific variables in future research to better elucidate the effects of BFRT in ACLR rehabilitation. Additionally, in the clinical application of BFRT, previous studies have highlighted the efficacy differences among various populations. Roman et al. [48] found that a standardized BFRT protocol significantly improved knee strength in adolescent ACLR patients. However, another study showed that, compared to standard rehabilitation methods, BFRT did not demonstrate superior efficacy in postoperative athletes with ACLR [49]. Therefore, the clinical application of BFRT may require further verification through studies and subgroup analyses targeting more diverse populations.

This meta-analysis has several limitations. Firstly, there were significant variations in patient characteristics (e.g., age, sex, postoperative timing), surgical methods (e.g., graft type), and BFRT intervention parameters (e.g., pressure levels, training intensity, and frequency) among the included studies. Such heterogeneity may impact the integration and interpretation of results, reducing the generalizability of the conclusions. The development of future clinical trials and adherence to standardized transplantation and BFRT intervention guidelines are crucial for rehabilitation recommendations for ACLR patients. Secondly, the follow-up periods in this study primarily focused on the early postoperative phase (≤ 3 weeks) and the mid-term (8–14 weeks), leaving the long-term effects of BFRT on muscle strength and knee function unassessed. Future research should extend follow-up durations to evaluate the long-term effects and potential sustained benefits of BFRT. Additionally, the overall sample size was relatively small, which may affect statistical power and limit further subgroup analyses. Larger-scale and higher-quality randomized controlled trials are needed to enhance the reliability of the findings.

## Conclusions

This study indicated that BFRT showed no significant advantages in early muscle strength recovery following ACLR but may have a positive impact on mid-term knee function, particularly in improving IKDC scores. However, due to heterogeneity and potential risk of bias in the included studies, the results should be interpreted with caution. Future high-quality research is needed to validate the long-term effects and safety of BFRT, providing stronger evidence to guide ACLR postoperative rehabilitation.

## Data Availability

No datasets were generated or analysed during the current study.

## Abbreviations

ACLR: Anterior Cruciate Ligament Reconstruction
ACSA: Anatomical Cross-Sectional Area
BFRT: Blood Flow Restriction Training
BMI: Body Mass Index
CI: Confidence Interval
IGF-1: Insulin-like growth factor
IKDC: International Knee Documentation Committee
MVIC: Maximal Volitional Isometric Contraction
PRISMA: Preferred Reporting Items for Systematic Reviews and Meta-Analyses
RCT: Randomized Controlled Trial
SMD: Standardized Mean Difference
